# Anxiety and Stress among American, Chinese, Italian, and Russian Emerging Adults: Does Uncertainty Avoidance Matter?

**DOI:** 10.3390/healthcare11243101

**Published:** 2023-12-05

**Authors:** Elisa Delvecchio, Giulia Cenci, Adriana Lis, Jian-Bin Li, Alexander T. Vazsonyi, Sofya Nartova-Bochaver, Magdalena Zadworna, Claudia Mazzeschi

**Affiliations:** 1 Department of Philosophy, Social Sciences and Education, University of Perugia, 06123 Perugia, Italy; giulia.cenci@unipg.it (G.C.); claudia.mazzeschi@unipg.it (C.M.); 2 Department of Developmental Psychology and Socialization, University of Padova, 35131 Padova, Italy; adriana.lis@unipd.it; 3 Department of Early Childhood Education, The Education University of Hong Kong, Hong Kong Special Administrative Region, Hong Kong, China; lijianbin@eduhk.hk; 4 Department of Family Sciences, University of Kentucky, Lexington, KY 40506, USA; vazsonyi@uky.edu; 5 Department of Psychology, National Research University Higher School of Economics (HSE University), Moscow 101000, Russia; s-nartova@yandex.ru; 6 Institute of Psychology, Faculty of Educational Sciences, University of Lodz, 90-128 Lodz, Poland; magdalena.zadworna@now.uni.lodz.pl

**Keywords:** anxiety, individualism/collectivism, cross-cultural, emerging adulthood, stress

## Abstract

Levels of anxiety and stress vary throughout the lifespan and across cultures. Uncertainty appears particularly relevant during emerging adulthood, thus potentially affecting both stress and anxiety. Uncertainty as a construct was identified by Hofstede (i.e., Uncertainty Avoidance Index, UAI), who defined it as the extent to which members of a culture feel threatened by ambiguous or unknown situations and tend to avoid them. The UAI was considered as a means to understand cultures in addition to the “classic” distinction between collectivist and individualistic cultures. The present study compared levels of anxiety and stress in 1790 university students (18–21 years old) from two individualistic (Italy and the US) and two collectivistic (China and Russia) countries, with a consideration of country UAI levels. Results showed that country-level UAI scores were associated with levels of anxiety and stress, controlling for age and sex. Italian and Russian students reported greater anxiety than American and Chinese ones. Chinese emerging adults reported the lowest stress levels, followed by American, Italian, and Russian students. The study findings provide initial evidence that anxiety and stress in emerging adults are associated with how a culture deals with perceived instability and uncertainty about the future.

## 1. Introduction

Anxiety and stress vary across the life course as well as across cultures. Over the past few decades, a growing body of literature has emerged that focuses on emerging adulthood [1,2], a specific developmental period starting during late adolescence and lasting through early adulthood. Although compared to adolescents, emerging adults (aged 18–30) are more independent and have the chance to explore various roles and life domains, they do not see themselves entirely as adults based on the belief that they have not yet fully formed individualistic qualities of character [1,2] such as self-sufficiency, self-responsibility, and independence in decision-making including financial and psychological independence. Like all transitional periods of life, emerging adulthood is an age of major opportunities for growth. However, it is also likely to be complicated by risks and challenges [3]. It is a period of great instability and uncertainty [1], often fraught with precarity and worry regarding one’s future, making young adults more vulnerable to mental health problems, including stress and anxiety disorders [3,4]. Both anxiety as well as stress are known to vary across the life course but also across different cultural contexts [5]. 

The term culture can be attributed to different nations, regions within nations, ethnic groups, occupations, and organizations. Different approaches for analyzing cultures have been proposed in research, and one of the most used classifications is Hofstede’s concept and model of national culture, as demonstrated by the large number of publications based on it [6,7]. Hofstede’s original model and the authors’ and colleagues’ more recent publication claim that the most important cultural issues can be captured by six cultural dimensions to which specific national indexes were calculated in different countries and made available at www.hofstede-insights.com (accessed on 20 February 2021): Individualism vs. Collectivism (*IDV*), Uncertainty Avoidance, (*UAI*), Power Distance (*PDI*), Motivation towards achievement and success (*MAS*), Long-Term Orientation (*LTO*), and Indulgence (IDG) (Value Survey Module, VSM) [8]. Hofstede’s six dimensions and indexes are the most widely recognized and robust framework for doing comparative research across cultures [9].

Concerning the possible relationships between culture and anxiety and stress in emerging adulthood, one dimension of Hofstede’s framework that looks of particular interest is UAI; it “can be defined as the extent to which the members of a culture feel threatened by uncertain or unknown situations” [10] (p. 191). How a society deals with uncertainty and an unknown future is the basic focus of UAI and appears to be of great significance in a stage in which individuals, such as emerging adults, need to develop and decide about their future lives, choices, and careers. Ambiguous or unknown situations and uncertainty about the future can be a source of worry, and it could increase anxiety and stress levels [11]. Should emerging adults try to control the future or just let it happen? Recent studies support the relationship between intolerance of uncertainty, anxiety, and stress, but mostly in clinical populations [12,13,14,15]. Only the study by Berenbaum et al. [12] highlighted how uncertainty was linked to worry, stress, and anxious arousal in a sample of college students. However, this study was carried out at the national level. Instead, in cross-national studies on anxiety and stress, attention was given to the role of individualism and collectivism variables (IDV) [16]. The country-level UAI index can add important information about levels of anxiety and stress in emerging adults from IDV countries. 

For this reason, the current exploratory study assessed the levels of anxiety and stress by comparing UAI and IDV across four countries (i.e., China, Italy, Russia, and the US). All these countries are located in the “high human development category” (UNDP, United Nations Development Program, 2018) with a high Human Development Index [17]. However, they show different UAI and IDV indexes, as derived from Hofstede’s VSM (2013) [8]. UAI and IDV use responses assessing, respectively, an individual’s sense of uncertainty in a group, which has a value between 0 (weak Uncertainty Avoidance) and 100 (strong Uncertainty Avoidance), and collectivism versus individualism between 0 (collectivism) and 100 (individualism). Russia’s UAI is 95, Italy scores 75 UAI, the US score is 46, and the Chinese UAI is 30. As for IDV, Italy and the US are considered individualistic societies (IDV score: 91 and 76, respectively), while Russia and China are considered collectivistic societies (IDV score: 39 and 20, respectively) [10,18].

According to Hofstede’s UAI score, individuals from China and the US share a “relaxed attitude” toward their future [19]. They did not feel threatened or uncertain due to an unknown future or ambiguity about life. This perhaps more “relaxed attitude” allows them to not experience high levels of anxiety and/or stress [19]. Chinese individuals grow up and live in a clear and well-defined context. Confucian doctrine and philosophy place great importance on putting the needs, cares, and interests of the family before one’s own [20]. Family, community, and society are central and prioritized in Chinese culture [21]. Moreover, the family and legal system also define the rules of marriage, the number of children, and future career choices. For this reason, many young people already have clear ideas about their future before their early twenties [21]. 

In the US, there is an ample degree of geographical mobility, whether it is technology or commerce, and this might support greater tolerance of different ideas or opinions [10]. American emerging adults have more opportunities for identity exploration of love, work, and worldviews compared to emerging adults and other cultural contexts [1,21]. They also grow up and live in such a socioeconomic context where they learn and need to know how to survive and cope using these opportunities. 

According to Hofstede’s UAI index, Russians and Italians share a feeling of being threatened or uncertain due to an unknown future or ambiguity about life because they share great economic challenges, instability, and a sense of the unknown about the future. Despite economic improvements, Russians experience feelings of uncertainty and confusion connected with the profound political, economic, and social instability due to changes that took place over the last twenty years, which have had a tremendous impact on the population, especially on younger generations, enhancing their levels of anxiety and stress [22]. 

As for Italy, emerging adults seem to experience greater difficulties in finding their place in society, and they experience high uncertainty and doubt about their future [23]. Data supports that about half of the emerging Italian adults still live at home with their parents, and one in three has not yet been emancipated from the family (https://ec.europa.eu/eurostat/web/products-eurostat-news/-/edn-20200812-1 accessed on 10 April 2021). Youth unemployment (18–24) is one of the highest in Europe (33%) [24]. Thus, emerging adults living in such a context are expected to show higher levels of anxiety and stress related to the future [25]. 

The current study aimed to support the hypothesis that differences in the level of anxiety and stress varied according to the UAI index and not IDV. In other words, Italian and Russian university students were expected to feel more threatened by ambiguous situations concerning their future and to show higher anxiety and stress levels than American and Chinese emerging adults. 

## 2. Materials and Methods

### 2.1. Sample

Convenience samples of 1760 university students (18–25 years old, mean age = 19.46, SD = 1.50; male = 30.5%) from four different countries: China (N = 538, mean age = 19.07, SD = 0.98; 39.2% males), Italy (N = 477; mean age = 21.17, SD = 0.98; 30.6% males), Russia (N = 329; mean age = 18.83, SD = 1.22; 25.2% males), and the United States (N = 446; mean age = 18.82, SD = 1.67; 24% males) were recruited for participation in the study. 

Each university was a public university that had students enrolled from across the country. They have a similar number of enrolled students every year and have a similar international rank in research and development compared with other universities in their country. Participants were enrolled mostly in social and behavioral science courses. Data were collected in all countries in the 2018/2019 academic year. The sample size was determined by considering the following factors: a significance level of 0.05; a power of 0.95; and a conservative small effect size. 

To carry out power analysis related to MANCOVA, 4 groups (i.e., 4 countries) and 2 response variables (i.e., anxiety, stress) were inserted. Since it is not possible to carry out a power analysis a priori for a MANCOVA, it was run considering each univariate effect (namely on anxiety and stress, separately) and covariates (i.e., sex and age). The analysis indicated that there was a 95% chance of correctly rejecting the null hypothesis of no difference between countries (4 groups), with a total sample of 1721 participants. Power analyses were carried out using G*Power 3.1 [26].

### 2.2. Measures

The Anxiety and Stress Scales of the Depression Anxiety Stress Scales-21 (DASS-21) is the short version of the Depression, Anxiety, and Stress Scales, a set of three self-report scales designed to assess the negative emotional states of depression, anxiety, and stress [27]. Each of the scales contains 7 items, rated on a 4-point severity/frequency scale, from “0 = did not apply to me at all” to “3 = applied to me very much, or most of the time”. The Anxiety scale assesses autonomic arousal, skeletal muscle effects, situational anxiety, and subjective experience of anxious affect (e.g., “I was worried about situations in which I might panic and make a fool of myself”). The Stress scale includes difficulty relaxing, nervous arousal, being easily upset/agitated, irritable/over-reactive, and impatient (e.g., “I found it difficult to relax”). Anxiety and Stress scales are calculated by summing the scores for the relevant items. A higher score indicates more severe negative emotions. The DASS-21 has been translated into several languages and showed adequate psychometric properties [5,28]. In this study, Cronbach’s α for the different countries were: for Anxiety, China 0.75 (95% CI [0.72, 0.78]), Italy 0.81 (95% CI [0.79, 0.84,]), the US 0.81 (95% CI [0.78, 0.83]), Russia 0.86 (95% CI [0.84, 0.88]); for Stress, China 0.76 (95% CI [0.73, 0.79]), Italy 0.85 (95% CI [0.82, 0.87]), the US 0.83 (95% CI [0.79, 0.84]), Russia 0.86 (95% CI [0.84, 0.88]).

### 2.3. Procedures

The administration of the survey was carried out in compliance with the ethical standards for research outlined in the 1964 Helsinki Declaration. This study received ethics review approval by the Ethics Committees or Institutional Review Boards at each university. Participants were informed about the study and provided their consent for participation, which was completely voluntary. No monetary reward or course credits were offered. Participants completed the anonymous questionnaires during regular class, and they had the right to discontinue the survey or to withdraw at any time.

### 2.4. Data Analysis

Mean anxiety and stress scores were compared across countries using Multivariate Analysis of Covariance (MANCOVA), with the country as a between-subject variable, controlling for age and sex. Results were considered significant at *p* < 0.05 with partial eta-squared > 0.01 [29]. Because multiple pairwise comparisons were carried out, a Bonferroni correction was employed, namely considering *p*-value < 0.008 as significant (i.e., 0.05/6 comparisons). Data analyses were carried out using SPSS (IBM SPSS Version 21).

## 3. Results

The MANCOVA provided evidence of a significant multivariate main effect for each country (Wilks’ λ = 0.74, *F*_(6, 3568)_ = 97.56, *p* < 0.001, *ŋp^2^* = 0.141) on both anxiety and stress. Age and sex, the covariates, had no significant effects (age Wilks’ λ = 1.00, *F*_(2, 1783)_ = 2.93, *p* = 0.053, *ŋp*^2^ = 0.00; sex: Wilks’ λ = 1.00, *F*_(2, 1783)_ = 0.12, *p* = 0.970, *ŋp*^2^ = 0.00). Means, standard deviations of each country, and Bonferroni post hoc comparisons are shown in Table 1.

**Figure 1:**
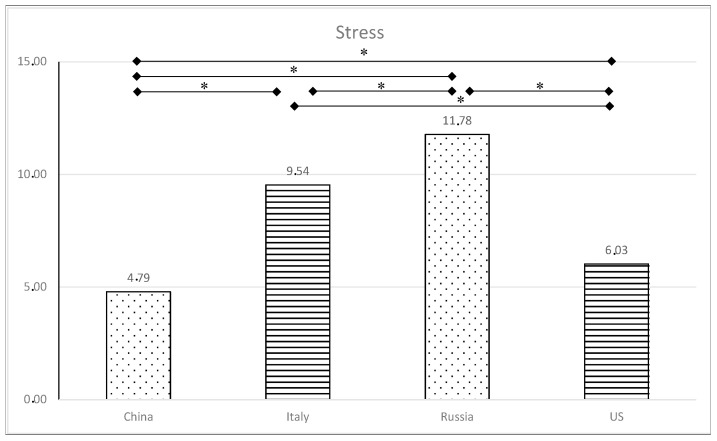
Levels of stress in the four countries. * p < 0.008.

**Figure 2:**
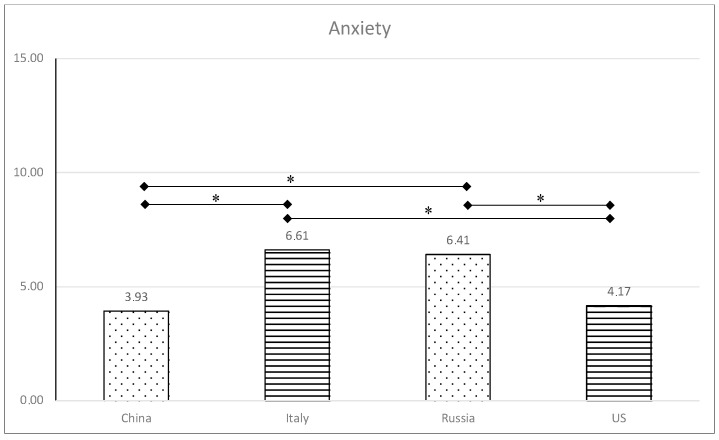
Levels of anxiety in the four countries. * p < 0.008.

Multiple post hoc comparisons following Bonferroni’s method provided evidence of differences in levels of anxiety and stress (see Figure 1 and Figure 2) by country. Specifically, anxiety scores from Italian and Russian emerging adults were significantly greater than those from Chinese and US students (*p* < 0.008).

For stress, significant differences were found across all countries: Russian emerging adults reported the highest levels of stress, followed by Italian, American, and Chinese adults.

## 4. Discussion

The current study examined how culture, namely the specific index of intolerance to uncertainty, was associated with two indicators of emerging adulthood’s well-being—anxiety and stress—in the pre-COVID-19 period. The key contribution of the present study concerned the examination of how Hofstede’s UAI country index, as a cultural indicator, was associated with levels of anxiety and stress in samples of emerging adults from both individualistic and collectivistic countries. Although emerging adulthood is a unique developmental period characterized by identity exploration and experimentation, in this period, individuals must deal with multiple tasks and challenges, which can cause a sense of instability and uncertainty, worries and concerns for precarity about their future, so it can represent a challenging and critical period for anxiety and stress [1,4]. However, ambiguity and uncertainty might modulate anxiety and stress differently across cultures [10,11]. Therefore, UAI seems to be a construct useful in trying to better understand potential similarities or differences across different cultural contexts.

Levels of stress and anxiety were compared in two individualistic and two collectivistic countries, as defined by Hofstede, known to differ in levels of UAI. Findings showed anxiety scores from Italian and Russian emerging adults were significantly greater than the scores from American and Chinese emerging adults. However, for stress, Russian emerging adults reported the highest levels of stress, followed by Italian, American, and Chinese emerging adults.

Russian emerging adults reported the highest levels of stress. This country had to make historical and political changes to overcome the previous basis connected with collectivism, but it seems to be unable to develop beliefs and institutions (like family and society) that try to avoid uncertainty and unknown situations. Thus, as expected, stress has a negative effect, indicating feeling upset or unpleasantly engaged, “a sense of high objective distress and encompassing a variety of affective states including upset, angry, guilty, afraid, sad, scornful and worried” reached a significantly higher level compared with the other countries examined [27,30]. Russian emerging adults also reported significantly more anxiety than American and Chinese emerging adults.

A different picture emerged for Italian emerging adults. They appeared significantly more anxious and stressed than American and Chinese emerging adults but less stressed than Russian emerging adults. The Italian UAI index, although high, was lower than the one in Russia. There are surely important differences concerning institutions in Italy. Although the political and social framework appears to provide only tentative support and leave emerging adults with a high sense of uncertainty, the family remains an important basic institution they can rely on. This may be one of the reasons why Italian emerging adults appear less stressed than anxious, meaning that they are more prone to situational anxiety, subjective experiences marked by anxious affect, somatic tension, and physical changes [27].

China and the US, although with very different cultural, historical, and political frameworks, appeared to have developed beliefs and institutions that help their members, specifically emerging adults, not feel threatened by uncertainty. Consequently, they appeared to be less stressed and anxious than Italian and Russian emerging adults. However, some differences appeared between the two countries concerning stress. American emerging adults appeared significantly more stressed than Chinese adults. As found by Nelson et al. [21], due to their beliefs and institutional context, many Chinese young people have clear ideas about their future before their early twenties, so it may be this awareness that allows them to perceive less stress. American emerging adults might need to challenge their future, and so they appeared significantly more stressed, but not at the same levels observed in the other two countries. The results of this study are consistent with previous research in clinical settings, which measured uncertainty at an individual level [14,15,16].

Therefore, UAI seems to be a useful construct in understanding different levels of anxiety and stress during emerging adulthood across different cultural contexts. Considering the different levels of uncertainty of one culture and the degree to which it tolerates ambiguity toward the future, it might represent a key issue to consider when studying anxiety and stress in both clinical and non-clinical settings.

This paper has several limitations that should be considered. First, the results of the present study lack generalizability due to sampling limitations. All participants were college students, so the results are limited and need to be replicated in different samples of emerging adults. Another limitation concerns the inclusion of only four countries. Furthermore, age and sex were only considered as covariates and not as focal variables. In fact, previous work by Mofateeh (2020) identified undergraduate students as being at greater risk than graduate students for anxiety and stress. However, the present study did not assess whether participants were undergraduates or graduate students, and thus, this question could not be addressed. As for sex, previous research has provided evidence that females are at greater risk of developing anxiety or stress than males [31]. Thus, future studies should further address this question rather than considering sex only as a covariate. Second, uncertainty avoidance was calculated through Hofstede’s model and not directly assessed from participants. Future studies might directly measure UAI using self-report tools. Moreover, only two dimensions of Hofstede’s model were considered. Future research might also benefit by examining within-culture differences, including education, ethnicity, and geographical regions. In addition, it would be important to assess cultural variables such as individualism–collectivism and uncertainty avoidance both at cultural and individual levels, also considering aspects of hierarchy and equality [20,32]. Additionally, future research also needs to more carefully consider how poverty or unemployment might be associated with the questions tested in the present study. Moreover, a number of additional, untested factors, in addition to UAI, might explain some of the observed differences highlighted in this study. Finally, the present study was correlational in nature and only focused on a single-dimensional construct (i.e., UAI).

## 5. Conclusions

In conclusion, the current findings provide useful information on the utility of considering the importance of specific cultural dimensions for the mental health of emerging adults. Specifically, uncertainty seems to play a key role in understanding the observed levels of stress and anxiety during emerging adulthood. Moreover, study findings offer a possible explanation for similarities and differences in levels of anxiety and stress during emerging adulthood, thus integrating both a cultural and developmental lens.

## Figures and Tables

**Table 1:** Descriptive summaries of DASS-21 scales by national group.

	China (N = 538)		Italy (N = 447)		Russia (N = 329)		USA (N = 446)		Bonferroni Post Hoc
	M	SD	M	SD	M	SD	M	SD	
Anxiety	3.93	3.34	6.61	4.74	6.41	4.76	4.17	3.89	C < I, R; USA < I, R
Stress	4.79	3.67	9.54	4.72	11.78	0.52	6.03	4.08	C < USA < I < R

## Data Availability

The data presented in this study are available on request from the corresponding author.

## References

[1] Arnett J.J. (2007). Emerging Adulthood: What Is It, and What Is It Good For?. Child Dev. Perspect..

[2] Arnett J.J. (2014). Presidential Address: The Emergence of Emerging Adulthood. Emerg. Adulthood.

[3] Lane J.A. (2015). Counseling Emerging Adults in Transition: Practical Applications of Attachment and Social Support Research. Prof. Couns..

[4] Ibrahim A.K., Kelly S.J., Adams C.E., Glazebrook C. (2013). A Systematic Review of Studies of Depression Prevalence in University Students. J. Psychiatr. Res..

[5] Zanon C., Brenner R.E., Baptista M.N., Vogel D.L., Rubin M., Al-Darmaki F.R., Gonçalves M., Heath P.J., Liao H.-Y., Mackenzie C.S. (2020). Examining the Dimensionality, Reliability, and Invariance of the Depression, Anxiety, and Stress Scale–21 (DASS-21) across Eight Countries. Assessment.

[6] Eringa K., Caudron L.N., Rieck K., Xie F., Gerhardt T. (2015). How Relevant Are Hofstede’s Dimensions for Inter-Cultural Studies? A Replication of Hofstede’s Research among Current International Business Students. Res. Hosp. Manag..

[7] Kaasa A., Vadi M., Varblane U. (2014). Regional Cultural Differences within European Countries: Evidence from Multi-Country Surveys. Manag. Int. Rev..

[8] Hofstede G., Minkov M. (2013). VSM 2013 Values Survey Module 2013 Manual Contents Page. http://www.laits.utexas.edu/orkelm/kelmpub/VSM2013_Manual.pdf.

[9] Gong W., Li Z.G., Stump R.L. (2007). Global internet use and access: Cultural considerations. Asia Pac. J. Mark. Logist..

[10] Hofstede G., Hofstede G.J., Minkov M. (2010). Cultures and Organizations: Software of the Mind: Intercultural Cooperation and Its Importance for Survival.

[11] Bakioğlu F., Korkmaz O., Ercan H. (2020). Fear of COVID-19 and Positivity: Mediating Role of Intolerance of Uncertainty, Depression, Anxiety, and Stress. Int. J. Ment. Health Addict..

[12] Berenbaum H., Bredemeier K., Thompson R.J. (2008). Intolerance of Uncertainty: Exploring Its Dimensionality and Associations with Need for Cognitive Closure, Psychopathology, and Personality. J. Anxiety Disord..

[13] Buhr K., Dugas M.J. (2009). The Role of Fear of Anxiety and Intolerance of Uncertainty in Worry: An Experimental Manipulation. Behav. Res. Ther..

[14] Carleton R.N., Mulvogue M.K., Thibodeau M.A., McCabe R.E., Antony M.M., Asmundson G.J.G. (2012). Increasingly Certain about Uncertainty: Intolerance of Uncertainty across Anxiety and Depression. J. Anxiety Disord..

[15] Mahoney A.E.J., McEvoy P.M. (2012). A Transdiagnostic Examination of Intolerance of Uncertainty across Anxiety and Depressive Disorders. Cogn. Behav. Ther..

[16] Knyazev G.G., Kuznetsova V.B., Savostyanov A.N., Dorosheva E.A. (2017). Does Collectivism Act as a Protective Factor for Depression in Russia?. Personal. Individ. Differ..

[17] United Nations Development Programme, UNDP (2018). Human Development Reports. https://hdr.undp.org/content/statistical-update-2018.

[18] Minkov M., Dutt P., Schachner M., Morales O., Sanchez C., Jandosova J., Khassenbekov Y., Mudd B. (2017). A Revision of Hofstede’s Individualism-Collectivism Dimension. Cross Cult. Strateg. Manag..

[19] Hofstede G. (1980). Culture and Organizations. Int. Stud. Manag. Organ..

[20] Li J.-B., Delvecchio E., Di Riso D., Salcuni S., Mazzeschi C. (2015). Self-Esteem and Its Association with Depression among Chinese, Italian, and Costa Rican Adolescents: A Cross-Cultural Study. Personal. Individ. Differ..

[21] Nelson L.J., Badger S., Wu B. (2004). The Influence of Culture in Emerging Adulthood: Perspectives of Chinese College Students. Int. J. Behav. Dev..

[22] Bochaver A.A., Hlomov K.D., Ali-zade A.S., Hlomov I.D., Kryukova T.L., Saporovskaya M.V., Hazova S.A. (2016). The future as a source of stress in modern teenagers. Psychology of Stress and Coping Behavior: Resources, Health, Development.

[23] Crocetti E., Tagliabue S., Sugimura K., Nelson L.J., Takahashi A., Niwa T., Sugiura Y., Jinno M. (2015). Perceptions of Emerging Adulthood. Emerg. Adulthood.

[24] Organisation for Economic Co-Operation and Development (2020). Unemployment—Youth Unemployment Rate—OECD Data.

[25] Haid M.-L., Seiffge-Krenke I., Molinar R., Ciairano S., Güney Karaman N., Cok F. (2010). Identity and Future Concerns among Adolescents from Italy, Turkey and Germany: Intra- and Between-Cultural Comparisons. J. Youth Stud..

[26] Faul F., Erdfelder E., Buchner A., Lang A.-G. (2009). Statistical Power Analyses Using G*Power 3.1: Tests for Correlation and Regression Analyses. Behav. Res. Methods.

[27] Lovibond P.F., Lovibond S.H. (1995). The Structure of Negative Emotional States: Comparison of the Depression Anxiety Stress Scales (DASS) with the Beck Depression and Anxiety Inventories. Behav. Res. Ther..

[28] Bibi A., Lin M., Zhang X.C., Margraf J. (2020). Psychometric Properties and Measurement Invariance of Depression, Anxiety and Stress Scales (DASS-21) across Cultures. Int. J. Psychol..

[29] Cohen J. (1988). Statistical Power Analysis for the Behavioral Sciences.

[30] Anderson E., Hope D. (2008). A Review of the Tripartite Model for Understanding the Link between Anxiety and Depression in Youth. Clin. Psychol. Rev..

[31] Mofatteh M. (2021). Risk factors associated with stress, anxiety, and depression among university undergraduate students. AIMS Public Health.

[32] Germani A., Delvecchio E., Li J., Mazzeschi C. (2020). Protective Factors for Depressive Symptoms in Emerging Adulthood. Scand. J. Psychol..

